# Macrolide Therapy in Chronic Inflammatory Diseases

**DOI:** 10.1155/2012/692352

**Published:** 2012-12-30

**Authors:** Kazuhito Asano, Elżbieta Tryka, Joong Saeng Cho, Naoto Keicho

**Affiliations:** ^1^Division of Physiology, School of Nursing and Rehabilitation Sciences, Showa University, Yokohama 226-8555, Japan; ^2^Department of Otolaryngology and Laryngological Oncology, Medical University of Lublin, Lublin 20-090, Poland; ^3^Department of Otolaryngology, School of Medicine, Kyung Hee University, Seoul 100-101, Republic of Korea; ^4^Department of Respiratory Diseases, Research Institute, National Center for Global Health and Medicine, Tokyo 162-0052, Japan

Sinobronchial syndrome is well accepted to involve the coexistence of chronic rhinosinusitis (CS) and chronic lower airway inflammation such as chronic bronchitis and diffuse panbronchiolitis (DPB). Although these diseases are resistant to several types of treatment, after discovery of the effectiveness of erythromycin on DPB, low-dose and long-term administration of macrolide antibiotics such as erythromycin, roxithromycin, and clarithromycin, are used frequently in the treatment of these diseases with remarkable success [1]. It has also reported that long-term use of azithromycin, a 16-membered macrolide antibiotic, can improve the lung functions in patients with cystic fibrosis (CF) [2]. These reports clearly indicate that the prognosis of these life-threatening airway diseases, especially DPB and CF, may improve dramatically, but the mode of action of this macrolide therapy is not fully understood. Furthermore, there is little information about the kind of diseases, which can be treated with the macrolide therapy. 

This special issue focuses mainly on 9 distinct papers to deal with the therapeutic mechanisms of macrolide on inflammatory diseases, the influence of macrolide antibiotics on respiratory viral infection, and the usefulness of macrolide therapy on inflammatory skin disease.

**Therapeutic Mechanisms of Macrolide Antibiotics**

Macrolides are a group of antibiotics with a macrocyclic lactone ring, which are classified into 14, 15, and 16 members, combined with sugar. These compounds are also accepted to be active against many species of Gram-positive and some Gram-negative bacteria and used frequently for the treatment of infectious diseases in respiratory tract. Besides their bacteriostatic and bactericidal effects, macrolides are used for the treatment of chronic airway inflammatory disease with remarkable success. However, the precise mechanisms by which macrolides could favorably modify the clinical status of chronic inflammatory diseases are not fully understood. B. Kwiatkowska and M. Maślińska and H. C. Steel review the therapeutic mode of action of macrolides on chronic inflammatory diseases. T. Shimizu and S. Shimizu examine the influence of azithromycin (AZ) on mucus hypersecretion in vitro and in vivo. They reveal the suppressive effects of AZ, but not josamycin and ampicillin on mucus secretion induced by inflammatory stimulation and propose that AZ will be a useful agent for the treatment of inflammatory diseases characterized by mucus hypersecretion. J. Bai et al. examine the influence of macrolide antibiotics on regulatory T-cell (Treg) functions through the choice of erythromycin and a rat model of smoke-induced lung inflammation (emphysema) and revel that oral administration of the agent into rat enhances Treg functions along with inhibition of lung inflammation. This novel data are very worthy to understand the therapeutic mode of action of macrolide antibiotics on airway inflammatory diseases.

**Respiratory Viral Infections**

The respiratory viral infections such as rhinovirus, respiratory syncytial virus, and influenza virus, among others, cause the high mortality rate through an overactive inflammatory response. Severity of airway viral infection is also accepted to be closely related with virus-induced hyperproduction of both inflammatory cytokines and chemokines, which are responsible for the development of fatal clinical symptoms such as massive pulmonary edema, acute bronchopneumonia, and acute respiratory distress syndrome. Since there is much evidence showing the suppressive effects of macrolide antibiotics on hyperproduction of inflammatory cytokines, macrolide antibiotics may be considered as promising treatment option in the treatment of airway viral infections. In this regard, J.-Y. Min and Y. J. Jang review the usefulness of macrolides in the treatment of airway viral infections. Furthermore, S. Yokota et al. also show the efficacy of macrolide antibiotics, especially clarithromycin, in the prevention of immunological disorders and secondary bacterial infections during airway viral infections.

**Skin Disorders and Bronchiectasis**

Long-term therapy with macrolide antibiotics is shown to be effective in the treatment of chronic airway inflammatory diseases such as CF, CS, and DPB. A. A. Alzolibani and K. Zedan and C. Rodrigues-Cerdeira et al. show the potential benefits of macrolide antibiotics in the treatment of cutaneous disorders such as atopic dermatitis, neutrophilic dermatitis, rosacea, and alopecia areata, among others. R. Masekala and R. J. Green also show the efficacy of macrolide antibiotics in the treatment of noncystic fibrosis-related bronchiectasis, an orphan lung disease, which results in impaired quality of life and mortality in paediatrics if it left untreated.
